# Piperacillin concentration in relation to therapeutic range in critically ill patients – a prospective observational study

**DOI:** 10.1186/s13054-016-1255-z

**Published:** 2016-04-04

**Authors:** Johannes Zander, Gundula Döbbeler, Dorothea Nagel, Barbara Maier, Christina Scharf, Mikayil Huseyn-Zada, Jette Jung, Lorenz Frey, Michael Vogeser, Michael Zoller

**Affiliations:** Institute of Laboratory Medicine, Hospital of the Ludwig-Maximilians-University of Munich, Marchioninistrasse 15, 81377 Munich, Germany; Department of Anaesthesiology, Hospital of the Ludwig-Maximilians-University of Munich, Marchioninistrasse 15, 81377 Munich, Germany; Max von Pettenkofer-Institute, Ludwig-Maximilians-University of Munich, Marchioninistrasse 17, 81377 Munich, Germany

**Keywords:** Target range, Intensive care unit, Creatinine clearance, Variability, Dosage, Antibiotics, Renal replacement therapy, C-reactive protein

## Abstract

**Background:**

Piperacillin levels after standard dosing have been shown frequently to be subtherapeutic, especially when renal clearance was augmented. Here, we aimed to determine if piperacillin was in its therapeutic range in a typically heterogeneous intensive care unit patient group, and also to describe target attainment dependent on daily dosage, creatinine clearance, and renal replacement therapy (RRT).

**Methods:**

Sixty patients with severe infections were included in this monocentric prospective observational study. Patients received 4.5 g of piperacillin-tazobactam two to three times daily by intermittent infusion depending on renal function according to clinical guidelines. Over 4 days, multiple serum samples (median per patient, 29; in total, 1627) were obtained to determine total piperacillin concentrations using ultra-high-performance liquid chromatography/tandem mass spectrometry.

**Results:**

A high heterogeneity of patient characteristics was observed (e.g., on day 1: creatinine clearance 2–233 mL/min and ten patients on RRT). Piperacillin trough levels showed inter-individual variation from 123 to >1785-fold on different study days. Each day, approximately 50 % and 60 % of the patients had piperacillin levels below the target ranges 1 and 2, respectively [defined for the calculated unbound piperacillin fraction according to the literature as 100 % time above MIC (100 %fT > MIC) (target range 1) and ≥ 50 %fT > 4 × MIC (target range 2); MIC = 16 mg/L]. Whereas only the minority of patients who received piperacillin-tazobactam three times daily (TID) reached target 1 (38 % on day 1), most patients who received piperacillin-tazobactam only twice daily (BID) because of severely impaired renal function reached this target (100 % on day 1). Patients with RRT had significant higher percentages of fT > MIC. Zero percent, 55 % and 100 % of patients without RRT who received antibiotics TID reached target 1 when creatinine clearance was > 65 mL/min, 30–65 mL/min and < 30 mL/min, respectively. In patients with causative strains only sensitive to piperacillin-tazobactam of all antibiotics given to the patient, piperacillin levels negatively correlated with CRP concentrations of day 4 (*p* < 0.05).

**Conclusions:**

A dosage of 4.5 g piperacillin-tazobactam TID seems to be frequently insufficient in critically ill patients, and also in patients where renal function is mildly to moderately impaired. For these patients, prescription of 4.5 g piperacillin-tazobactam four times daily could be considered.

**Trial registration:**

Clinicaltrials.gov NCT01793012. Registered 24 January 2013.

**Electronic supplementary material:**

The online version of this article (doi:10.1186/s13054-016-1255-z) contains supplementary material, which is available to authorized users.

## Background

Severe infections are a leading cause of the observed high morbidity and mortality in intensive care unit (ICU) patients. A key element for optimal patient outcomes includes sufficiently high antimicrobial concentration levels [1]. Such concentrations are also important for preventing the development of antimicrobial resistance [2, 3].

Piperacillin (PIP) is one of the most commonly used antibiotics in ICUs [4, 5]. This hydrophilic drug, which is predominantly excreted renally, is often prescribed together with the beta-lactamase inhibitor tazobactam (TAZ) as a fixed combination. After intermittent administration, PIP concentrations varied substantially among critically ill patients [6–10]. However, most studies have evaluated only limited numbers of patients or defined patient subgroups, resulting in inconclusive information regarding just how substantial the inter-individual variation of PIP concentrations is in a typical heterogeneous ICU patient group. Recent studies have been performed with high patient numbers [11–13], but limited numbers of PIP concentrations were determined per patient. Indeed, there is only one report concerning PIP concentrations over an entire 7-day antibiotic course in critically ill patients [6]; the 11 patients included in that study exhibited coefficient of variations (CV) of 20–60 % for within-patient variability (for trough levels). Because this variability was evaluated only in patients with normal renal function, it remains unclear if this result reflects the typical intra-individual variability within ICU patients.

It has been shown that the high variability of piperacillin levels observed in ICU patients often leads to potentially subtherapeutic levels. This is mostly dependent on renal function; therefore – according to expert information and guidelines – different dosage schemes are recommended depending on creatinine clearance. Udy et al. recently evaluated PIP levels in dependence of creatinine clearance in critically ill patients with septic shock/severe sepsis receiving 4.5 g PIP-TAZ four times daily by intermittent short-term infusions. In patients grouped by creatinine clearance quartiles, most patients showed potentially insufficient PIP trough levels when creatinine clearance was 115–170 mL/min or > 170 mL/min [14]. Administration of 4.5 g PIP-TAZ three times daily (TID) was also shown frequently to result in insufficient levels in critically ill patients with moderately impaired renal function [15]; however, this was evaluated in the early phase of treatment, and it is not clear if this result is transferable to later therapy time points. Indeed, many physicians still usually prescribe only 4.5 g PIP-TAZ TID for critically ill patients, especially for patients with mildly or moderately impaired renal function, and 4.5 g PIP-TAZ twice daily (BID) in cases of severely impaired renal function. Some authors assumed that the usual reduction of the piperacillin dosage especially for patients with severely impaired renal function might be critical in case of serious illness. Indeed, according to the German guideline “Epidemiology, diagnosis and treatment of adult patients with nosocomial pneumonia,” critically ill patients, in particular with a reduced dose because of impaired renal function, might be underdosed [16]. Therefore, it is recommended here that the first dose should not be adapted in patients with septic shock/severe sepsis with severely impaired renal function. However, studies describing PIP levels with such a dosage regimen (4.5 g PIP-TAZ BID) in critically ill patients are still lacking.

Therefore, we designed a prospective observational study to analyze the variability of PIP in a patient group of critically ill patients. The aim was to determine if PIP was in its therapeutic range in a typically heterogeneous ICU patient group and also to describe target attainment dependent on daily dose, creatinine clearance, and renal replacement therapy (RRT).

## Methods

### Patients

Sixty consecutive patients who met the inclusion criteria and who were hospitalized from September 2013 to September 2014 in one of three ICUs within the Department of Anaesthesiology, University Hospital of Munich, were included. The inclusion criteria consisted of the presentation of severe infection (confirmed or clinically assumed) and the therapeutic intravenous administration of PIP-TAZ via short-duration infusions. The exclusion criteria were age < 18 years, a planned hospitalization time of < 4 days and the administration of PIP-TAZ more than 48 hours before the study began. Written informed consent for study inclusion was obtained from all patients or their legal representatives.

### Study design

The study protocol (NCT01793012) for this prospective observational study was approved by the ethics committee of the Ludwig Maximilians University and was performed in accordance with the ethical standards set forth by the 1964 Declaration of Helsinki and its later amendments. Patients received 4.5 g of PIP-TAZ BID or TID depending on renal function according to clinical guidelines. The beginning of the study (day 1) was defined as the time point at which the first blood sample was taken from each patient to determine PIP concentrations. Serum samples for antibiotic determination were collected at multiple time points immediately before (trough level), during, and after all PIP-TAZ administrations on day 1 (sampling after the start of PIP-TAZ administrations at 0.25, 0.5, 1.5, 4, 7.25 or 8, and 12 hours, if appropriate). On days 2, 3, and 4, samples were collected during only one of the PIP-TAZ intervals (sampling at the same time points). The exact time of blood sampling was recorded by the medical staff. Serum samples for determination of PIP levels were immediately (<15 minutes) sent by pneumatic delivery system to the Institute of Laboratory Medicine. There, samples were immediately centrifuged, aliquoted, stored within 1 hour after sampling at -20 °C, and finally, within 24 hours after sampling, at -80 °C.

### Laboratory and clinical parameters

Total PIP concentrations were determined using a two-dimensional ultra-high-performance liquid chromatography/tandem mass spectrometry (UHPLC-MS/MS) method as previously described [17]. Five-fold deuterated piperacillin was used as the internal standard. Validation revealed good analytical performance with an inaccuracy ≤ 5 % and imprecision ≤ 5 % (CV) for all quality control samples. For renal function evaluation, 24-hour urine was collected and creatinine clearance (CL_crea_) was calculated using the formula:

$$ {\mathrm{C}\mathrm{L}}_{\mathrm{crea}}=\left({\mathrm{C}}_{\mathrm{urine}}*{\mathrm{V}}_{\mathrm{urine}}\right):\left({\mathrm{C}}_{\mathrm{serum}}*\mathrm{time}\right), $$

where C_urine_ is the creatinine concentration in urine, V_urine_ is the urine volume, and C_serum_ is the serum creatinine concentration. Diagnosis, laboratory and clinical parameters/scores were recorded daily in the ICUs.

### Strains

Pathogens isolated from the patients between 6 days before and 6 days after the study began were recorded. Susceptibility to PIP-TAZ for bacteria was determined in accordance with the breakpoints as described by the European Committee on Antimicrobial Susceptibility Testing [18].

### Correlation of outcome parameters with PIP concentrations

Outcome parameters (mortality within 28 days and ICU-free days on day 28) and C-reactive protein (CRP) levels were evaluated in all patients and in a subgroup of patients defined as outcome group. In this group, only patients with bacterial isolates that met all the following criteria were included: (1) the isolates were clinically assumed to be causative strains for the patients’ infection, (2) all assumed causative strains were bacteria and were sensitive to PIP-TAZ, (3) but not to other antibiotic substances given to the patient. PIP trough levels of day 1 were correlated with alive ICU-free days on day 28 and with 28-day mortality. For CRP, we correlated PIP trough levels with CRP concentrations and with changes of CRP (CRP quotients). Finally, we correlated PIP trough levels of day 1 with possible neurotoxic effects within the study period (patients with delirium, somnolence, or seizures within the study period) in all patients.

### Assessment of target concentration ranges

The thresholds for the potential therapeutic efficacy of total PIP were defined as > 22.5 mg/L for trough levels (target range 1) and/or a percentage of time > 90 mg/L (%T > 90 mg/L) of ≥ 50 % (target range 2). The 22.5 mg/L trough level corresponds to a percentage of time above the minimal inhibitory concentration (%T > MIC) of 100 % [13, 14] using a MIC value of 16 mg/L [6, 14, 18] and taking into account an average protein-binding fraction of 30 % [12, 19–22]. Analogously, %T > 90 mg/L of ≥50 % corresponds to a %T > 4 × MIC of ≥50 % in accordance with the literature [19, 23].

### Pharmacokinetic analysis

PIP trough levels, as measured using UHPLC-MS/MS, were evaluated. For the determination of the percentage of time the PIP concentrations remained above 90 mg/L or 22.5 mg/L, we connected the different determined PIP concentration points between two consecutive PIP-TAZ administrations and determined the time point at which the PIP concentration fell below 90 mg/L or 22.5 mg/L, respectively. The time between the beginning of the previous PIP-TAZ administration and this time point was set in relation to the entire time between the two respective PIP-TAZ administrations.

### Statistics

The boxplots represent the medians and interquartile ranges, and the ends of the whiskers represent the 5th and 95th percentiles. The intra-patient variability CV was calculated by dividing the individual standard deviation by the mean of a given patient’s trough levels. The Spearman correlation coefficient was used to describe the correlations between continuous parameters. The Wilcoxon-Mann-Whitney test was used to test significance between different groups.

## Results

The beginning of the sampling was 6.9–45.6 hours (median, 18.7 hours) after the first PIP-TAZ administration. Over the 4-day study, multiple blood samples (median, 29; interquartile range (IQR) 20–33) were taken from each patient. The 60 patients exhibited a high heterogeneity in clinical and laboratory characteristics (Table 1). The most frequent causes of infection were pneumonia (*n* = 36) and peritonitis (*n* = 7). Twelve patients suffered from acute respiratory distress syndrome. Seven patients were post-lung, and eight patients were post-liver transplant patients. Three patients were treated with extracorporeal lung assist, and ten patients were treated with renal replacement therapy (RRT) (day 1). In total, 33 gram-positive bacterial strains, 19 gram-negative bacterial strains, 23 fungal and six viral strains were detected in the patients.

**Table 1:** Clinical and demographic characteristics of included patients

Characteristic^a^	All patients	Patients with 4.5 g PIP/TAZ three times daily	Patients with 4.5 g PIP/TAZ two times daily
	(n = 60)	(n = 45)	(n = 15)
Female/male (n/n)	17/43	13/32	4/11
Age (years)	63 (54–75)	63 (55–74)	64 (54–78)
BMI (kg/m^2^)	27 (24–29)	26 (23–29)	29 (26–31)
Severity scores			
APACHE II score	24 (18–31)	24 (17–32)	24 (18–29)
SOFA score	11 (9–14)	11 (9–13)	12 (11–17)
Laboratory values			
CrCl (mL/min)	60 (24–119)	70 (40–130)	11 (6–19)
Organ transplantation (n)			
Lung	7	6	1
Liver	8	4	4
Special treatments (n)			
RRT patients	10	7	3
ECLA	3	3	0
Site of infection (n)			
Pneumonia	36	27	9
Peritonitis	7	4	3
Catheter associated	5	4	1
Others	12	10	2
Presence of ARDS (n)	12	11	1

*PIP/TAZ* piperacillin-tazobactam, *BMI* body mass index, *APACHE II* Acute Physiology and Chronic Health Evaluation II, *SOFA* Sequential Organ Failure Assessment, *CrCl* creatinine clearance of patients without renal replacement therapy, *RRT* renal replacement therapy, *ECLA* extracorporeal lung assist, *ARDS* acute respiratory distress syndrome

^a^Data from day 1 are presented as median (interquartile range) unless otherwise specified

**Fig. 1:**
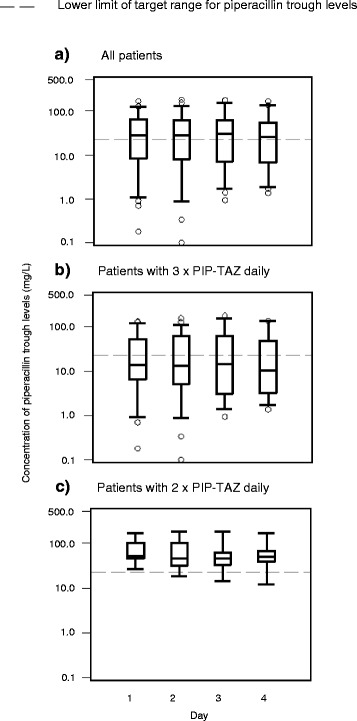
Distribution of piperacillin trough levels over the course of 4 days. Trough levels **a** for all patients **b** for patients receiving piperacillin-tazobactam (PIP-TAZ) three times daily, and **c** for patients receiving PIP-TAZ two times daily because of impaired renal function are shown. Boxplots represent medians and interquartile ranges, and the ends of the whiskers represent the 5th and 95th percentiles

A total of 1627 PIP concentrations were included for evaluation. Additional file 1 shows the concentration time curves of serum PIP concentrations in all patients. A high inter-individual variability for PIP was observed. This result was not restricted to the beginning of the study period (range of trough levels on day 1: 928-fold, 0.18–167 mg/L) but was observed over 4 days (range of trough levels 123-fold to > 1785-fold at each day 2, 3, and 4) (Fig. 1a). The PIP concentration variability was similarly substantial in patients who received PIP-TAZ TID (range of trough levels 99-fold to > 1549-fold on the different study days) (Fig. 1b), a patient group characterized by higher creatinine clearance (Table 1). In contrast, less variability was observed in the PIP concentrations of patients who received PIP-TAZ only BID (range of trough levels 6.2-fold to 13.8-fold on the different study days) (Fig. 1c), a patient group characterized by the presence of strongly impaired renal function.

Intra-patient variability was much lower than inter-patient variability. Over 4 days, only four patients exhibited maximum trough levels greater than 10-fold higher than the minimum trough levels. Most of the 60 patients exhibited values either always (n = 22) or partly below (n = 14) the target threshold. CVs for within-patient PIP variability ranged from 6.4 to 129 % (median, 30 %; IQR, 23–44 %). The PIP trough level of most patients did not change in a consistent pattern over the 4 days of the study; only in patients 15, 17, 25, 45, and 51 we did observe a continuous decrease, and in patient 59 a continuous increase of PIP trough levels over time (Additional file 1).

Approximately 50 % of all study patients exhibited PIP levels below target range 1 (trough levels < 22.5 mg/L) (Table 2). Over 4 days, adequate trough levels of PIP were observed in almost all patients from the patient group with the lowest quartile of creatinine clearance (100 % on day 1). In contrast, adequate PIP trough levels were not observed in the patient group with the highest quartile of creatinine clearance over the course of 4 days. Similarly, adequate PIP trough levels were reached by almost all patients who received PIP-TAZ BID because of impaired renal function (100 % on day 1; IQR of creatinine clearance: 6–19 mL/min), whereas this target was only reached by the minority of patients receiving PIP-TAZ TID (38 % for PIP on day 1; IQR of creatinine clearance: 40–130 mL/min). Indeed, in patients who received PIP-TAZ TID (patients on RRT excluded), no patient with a creatinine clearance > 65 mL/min, and only 55 % of the patients with a creatinine clearance ranging from 30 to 65 mL/min, reached this target (Additional file 2). In contrast, patients with equal antibiotic dosages and a creatinine clearance < 30 mL/min always had piperacillin levels within the therapeutic range. The dependency of trough levels on creatinine clearance was proven (r = -0.837) (*p* < 0.001).

**Table 2:** Distribution of patients in relation to the target ranges of piperacillin

Patient groups, number of patients^a^	Percentages of patients who attain the targets			
	trough values ≥ 22.5 mg/L		≥50 % of time > 90 mg/L	
	Day 1	Day 4	Day 1	Day 4
Total patients, n = 60	53 %	52 %	40 %	38 %
With lowest quartile^b^ of CrCl, n = 11	100 %	86 %	62 %	67 %
With highest quartile^c^ of CrCl, n = 11	0 %	0 %	9 %	0 %
With RRT, n = 10	80 %	71 %	62 %	57 %
Without RRT, n = 50	48 %	49 %	36 %	33 %
Receiving 2 × 4.5 g PIP/TAZ daily, n = 15^d^	100 %	82 %	64 %	50 %
Receiving 3 × 4.5 g PIP/TAZ daily, n = 45^e^	38 %	35 %	33 %	32 %

*CrCl* creatinine clearance, *RRT* renal replacement therapy, *PIP/TAZ* piperacillin-tazobactam

^a^Numbers as determined on day 1 are presented

^b^Creatinine clearance 2–19 mL/min

^c^Creatinine clearance 108–233 mL/min

^d^Comprising on first study day three patients with RRT and 12 patients without RRT. The latter patients presented a median creatinine clearance of 11 mL/min (interquartile range: 6–19 mL/min)

^e^Comprising on first study day seven patients with RRT and 38 patients without RRT. The latter patients presented a median creatinine clearance of 70 mL/min (interquartile range: 40–130 mL/min)

Similar observations with respect to target attainment were made for the specific patient subgroups when using target range 2 (≥50 %T > 90 mg/L); however, fewer patients of the patient subgroups were in the therapeutic range (e.g., approximately 40 % of total patients over 4 days) (Table 2, Additional file 3). Whereas the difference between target attainment of target range 1 and 2 was low for patients who received PIP-TAZ TID (38 % versus 33 % on day 1, respectively), a high difference was observed for patients who received PIP-TAZ BID (100 % versus 64 %) (Table 2).

**Fig. 2:**
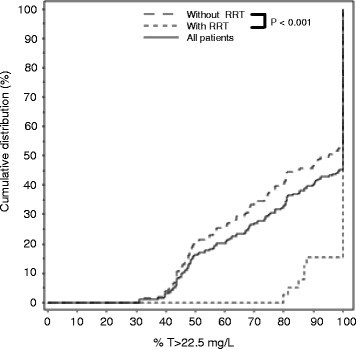
Effects of renal replacement therapy on percentage of time > 22.5 mg/L of piperacillin. Percentage of time of piperacillin (PIP) > 22.5 mg/L for values of all patients, for values of patients with renal replacement therapy (RRT), and for values of patients without RRT are shown. From each patient, one value was evaluated per day, and values of the 4 days of all patients were evaluated simultaneously

Over 4 days, adequate trough levels of PIP were observed in most patients with RRT (80 % on day 1) (Table 2). Indeed, presence of RRT [different daily dosages (BID or TID) and different RRT methods used, see Additional file 4] was associated with significant higher percentages of T > 22.5 mg/L (Fig. 2) (*p* < 0.001). Target attainment of the different subgroups in dependence of dosing, creatinine clearance, and use of RRT are shown in Additional file 5.

PIP trough levels had no significant effects on both mortality and ICU-free days on day 28 in all patients. Within 28 days, six patients died, 40 patients survived and 14 patients were discharged from hospital. The median of ICU-free days at day 28 was 14 (IQR 0–23). We further analyzed the outcome group as defined in Methods: also here, no significant effects of PIP trough levels on both mortality and ICU-free days on day 28 were observed. Finally, we evaluated if PIP trough levels might have at least an effect on the course of inflammation parameters within 4 study days. In the outcome group, we observed a significant negative correlation between CRP concentrations on day 4 versus PIP trough levels over time (Table 3). A similar trend for day 3 and 4 was observed when quotients of CRP concentrations were correlated with PIP trough levels over time.

**Table 3:** Analysis of outcome group^a^

Correlation of piperacillin trough levels^b^ with CRP levels	Number of patients	Correlation coefficient	*p* value
Trough level day 1 versus CRP day 1	13	0.13	0.67
Trough levels day 1 + 2 versus CRP day 2	13	- 0.22	0.94
Trough levels day 1 + 2 + 3 versus CRP day 3	12	- 0.31	0.33
Trough levels day 1 + 2 + 3 + 4 versus CRP day 4	11	- 0.66	0.028^*^
Trough levels day 1 + 2 versus the quotient CRP day 2/1	13	- 0.12	0.71
Trough levels day 1 + 2 + 3 versus the quotient CRP day 3/1	12	- 0.50	0.095
Trough levels day 1 + 2 + 3 + 4 versus the quotient CRP day 4/1	11	- 0.55	0.083
Pathogens of the outcome group	Number of pathogens		
*Burkholderia multivorans*	1		
*Citrobacter freundii*	1		
*Enterococcus faecalis*	3		
*Klebsiella oxytoca*	1		
*Klebsiella pneumoniae*	1		
*Proteus mirabilis*	1		
*Propionibacterium acnes*	3		
*Pseudomonas aeruginosa*	2		
*Pseudomonas oleovorans*	1		
*Raoultella ornithinolytica*	1		
*Staphylococcus aureus*	4		
Streptococci beta hemolytic group C	1		
*Streptococcus anginosus*	1		
*Streptococcus pneumoniae*	1		
*Streptococcus viridans*	1		

*CRP* C-reactive protein

^a^In this group, only patients with bacterial isolates that met all the following criteria were included: (1) the isolates were clinically assumed to be causative strains for the patients’ infection, (2) all assumed causative strains were bacteria and were sensitive to PIP-TAZ, (3) but not to other antibiotic substances given to the patient

^b^Only one piperacillin trough level used per day

Finally, there was no difference in the presence of PIP trough concentrations on day 1 and possible neurotoxic effects over the study period.

## Discussion

In our ICU patient group, we observed a very high variability of PIP levels leading to a high percentage of patients with potentially subtherapeutic PIP concentrations. Target attainment was tightly associated with creatinine clearance. Although the strong association between creatinine clearance and piperacillin levels has already been described in several studies of critically ill patients, this work is novel in contemporary literature, given the number of blood-sampling time points, the variability of piperacillin levels observed over several days, the difference of target attainment dependent on both creatinine clearance and commonly used dosage schemes, and the increasing negative association of PIP trough levels with CRP levels during therapy. Our data highlights the concern that patients with mild or moderate renal function impairment are also likely to have subtherapeutic levels over several days with the conventional intermittent dosage scheme of 4.5 g PIP-TAZ TID. This might lead to treatment failure or the selection of drug-resistant strains. In contrast, there might be less risk of subtherapeutic trough levels and treatment failure in ICU patients with severely impaired renal function and use of RRT, even when the dosage is reduced to 4.5 g PIP-TAZ BID.

Previous studies have also described a high dependency of PIP concentrations on creatinine clearance [14, 15, 24–29]. However, only Conil et al. described the effect on target attainment when using this conventional dosing in patients with slightly to moderately impaired renal function [15]. They observed a correlation coefficient of -0.61, whereas we observed an even higher correlation (r = -0.837) for patients receiving 4.5 g PIP-TAZ TID. Because Conil et al. determined PIP concentrations at precisely the time point of 24 hours after starting antibiotic treatment, it remained unclear if the observed high percentage of subtherapeutic levels in their study might also have resulted from insufficient loading and how often the target range would be attained at a later time points. The high correlation coefficient we found may open the possibility to develop a pharmacokinetic model, which might help to find adequate dosing especially in dependence of creatinine clearance values. We showed that the percentage of insufficient levels remained high and more or less stable in patients over the course of several days. Moreover, all patients with a creatinine clearance > 65 mL/min and approximately half of patients with a creatinine clearance between 30 and 65 mL/min had insufficient levels, even with the lower therapeutic range (therapeutic range 1). This shows that this dosage may be insufficient over several days also for patients who have slightly or moderately impaired renal function. To reach the target range 1, it might be reasonable in cases of slightly to moderately impaired renal function to prescribe the maximum dosage of 4.5 g PIP-TAZ four times daily, as recommended by the prescription drug information. However, occurrence of neurotoxicity, although not clearly observed in our study, should be carefully monitored. Moreover, to the best of our knowledge, we showed for the first time that an adjusted PIP dosage of 4.5 g PIP-TAZ BID for patients with strongly impaired renal function leads to therapeutic levels in critically ill patients much more often (Table 2). Interestingly, for these patients, this was much more dependent on the defined therapeutic range (1 versus 2). Whereas most patients reached therapeutic range 1 (82–100 % at different study days), only 50–64 % reached therapeutic range 2 (Table 2). This might be due to the longer time intervals between consecutive doses in this case, which makes it more difficult to reach a high concentration over a long time interval. There is still a controversial debate about the right target range for critically ill patients [13]. However, the high percentage of patients with severely impaired renal function and reduced dosage reaching at least the target range 1 shows that the problem of underdosing in this patient group is much lower than in patients with slightly or moderately impaired renal function and not an adjusted dosage. This is still important, because many ICU physicians still prescribe PIP-TAZ 4.5 g TID – at least for patients with slightly to severely impaired renal function. Indeed, these dosages are still recommended by the Food and Drug Administration and in expert information.

We also evaluated the influence of RRT on PIP concentrations. This is important as the use of RRT may further alter antibiotic pharmacokinetics [30]. We observed significant higher percentages of T > 22.5 mg/L for the patients with RRT. Because of the different RRT methods and the different daily dosages, we only had limited numbers of the specific subgroups. Therefore, we could not evaluate the influence of the different RRT modalities on PIP concentrations.

Considering all patients, we observed a high variability and a high quantity of subtherapeutic levels for PIP, as in other studies [6, 9, 19, 21, 31, 32]. In contrast with the literature, we observed an even higher inter-individual variability for PIP trough levels [6, 9, 19, 21, 33] and a higher percentage of patients with PIP levels below fT > MIC [6, 9, 14] and below the higher target range of 50 % fT > 4 × MIC [9, 19, 23]. Sime et al., however, observed a higher percentage of patients with trough levels below the target range [21], which might be due to the average higher creatinine clearance (all patients > 50 mL/min) of their study patients. The high inter-individual variability we observed in our study with trough levels varying > 100-fold was restricted to patients who received PIP-TAZ TID (Fig. 1), which might be at least in part due to the higher range of creatinine clearance in this group. We also found partly higher intra-patient variability than in the literature: over 4 days, we observed CVs for PIP ranging from 6 to 129 %, whereas Carlier et al. reported CVs of 20–60 % over an entire antibiotic course [6]. The reason for the higher variability observed in our study might be the higher heterogeneity of patient characteristics. As this is typical for ICU patients, our data might support the concept of therapeutic drug monitoring (TDM).

Thresholds of both target ranges for unbound PIP were defined as in other studies [6, 9, 14, 19, 21, 23]. A traditional target for this antibiotic is 40–50 %fT > MIC [34]. However, a higher target range might be more appropriate for ICU patients [31] because of the critical illnesses of these patients. Therefore, we chose 100 %fT > MIC in accordance with most other studies [6, 9, 14, 21]. Indeed, different studies demonstrated that for beta-lactam antibiotics, maximum killing is often only achieved when the T > MIC approaches 90–100 % of the dosing interval [35]. The higher target range (50 %fT > 4 × MIC) was chosen in accordance with other studies [19, 23], because antibacterial killing of beta-lactam antibiotics might be maximal when the antibiotic is 4–5 × MIC [36]. A long time-interval above such a higher threshold might also be positive to reduce the development of antibiotic resistance [3]. However, it should be noted that even higher target ranges (i.e., 100 %fT > 4 × MIC) might be useful to maximize the antimicrobial effect and minimize the development of resistance in these patients [3, 19]. Target attainment might also vary in dependence of microorganism idiosyncrasies such as inoculum [37]. Future prospective interventional studies are required to investigate which target ranges are associated with the best outcome for patients, thereby also minimizing the risk for the development of antibiotic resistance in the intensive care unit.

Outcome parameters such as alive ICU-free days, 28-day mortality, or occurrence of adverse reactions did not correlate with PIP levels in all patients. However, because of the patient number and the heterogeneous patient group used, short-term effects of antimicrobial therapy on alive ICU-free days and 28-day mortality might not be visible in this group. We therefore also correlated CRP concentrations in both all patients and in the outcome group. In the outcome group, we found a negative correlation of CRP concentrations at day 4 with PIP trough levels (*p* < 0.05). Furthermore, a trend to a faster CRP decrease in cases of high PIP trough levels was also observed at day 3 and 4 (both *p* < 0.1, Table 3). We thought that it might be important to define an outcome group, because we wanted to evaluate only relevant patients, where PIP-TAZ was the only antimicrobial therapy effective against the causative pathogens. As it has not yet been shown in the literature for piperacillin that target attainment of our targets correlates with a positive clinical or microbiological outcome, the targets used in our study have to be regarded only as a limited approach. However, our data indicate that at least higher concentrations of PIP might be associated with a faster decrease of the inflammation parameter CRP.

This study has some limitations. (1) The data were drawn from a single center. Indeed, PIP pharmacokinetics might be different among patients from different ICUs. To minimize this limitation, we included a relatively high number of patients and allowed a high heterogeneity of patient characteristics to best represent the full spectrum of different patients in ICUs. (2) We did not measure the unbound fraction of PIP from stored serum samples, e.g., via centrifugal filter devices. Indeed, it remains unclear if PIP might be bound to the filter in a relevant percentage and if freeze-thaw cycles might alter the protein-binding fraction. However, we considered a fixed protein-binding fraction of 30 % in the definitions of the target ranges. This may be a problem in individual patients, because unbound PIP fractions may differ substantially in individual critically ill patients, especially in those with altered protein levels. (3) Higher thresholds of the target ranges were not defined, as no robust data are existent. However, definition of such thresholds may also be important because it has been shown that the neurotoxicity of some beta-lactam antibiotics may be probably underestimated in critically ill patients [38]. (4) Only two different dosing schemes were evaluated. Some authors have used higher doses for critically ill patients [6, 14]; however, the dosage schemes used in our study are still recommended by the Food and Drug Administration and in expert information. Prolonged or continuous infusions have also been recommended [34]. Indeed, it has been shown by Abdul-Aziz et al. that prolonged infusion of piperacillin can be associated with a better outcome [39] but no difference in the clinical outcome was observed in other studies [40, 41]. Moreover, some authors also write that a prolonged or continuous infusion eventually promotes that resistant mutants are selectively amplified, and that these mutants therefore might become the dominant bacterial population [2] (5) Finally, we did not use pharmacokinetic modeling to describe %T > 90 mg/L or trough levels. Such models are especially relevant if only few samples per patient are available or if concentrations around the peak value have to be described. We did not perform pharmacokinetic modeling because we collected multiple blood samples between consecutive antibiotic administrations (Additional file 1), and we only described the %T > 90 mg/L and the trough levels, which were always on the descending concentration time curves. Indeed, imprecise individual predictions might occasionally occur with pharmacokinetic modeling if the individual estimated parameter values vary substantially from those of the typical population, which might be a problem in a highly heterogeneous patient group as in our study [42].

## Conclusions

Our data emphasize that the conventional dosing of 4.5 g PIP-TAZ TID may often lead to insufficient blood levels in critically ill patients, and also in patients with slightly or moderately impaired renal function. In contrast, underdosing might be less common in patients with severely impaired renal function or in patients with RRT, also in cases of 4.5 g PIP-TAZ BID.

## Key messages

- A high inter- and intra-individual variability of piperacillin levels was observed leading to a continuously high percentage of potentially subtherapeutic levels over several days during the acute phase of severe infections.
- Piperacillin trough levels were tightly associated with creatinine clearance.
- Only the minority of patients who received PIP-TAZ 4.5 g TID had trough levels for the calculated unbound fraction of piperacillin > 16 mg/L (target range 1), whereas most patients who received PIP-TAZ 4.5 g only BID because of severely impaired renal function attained this target range.
- Use of renal replacement therapy led to significant higher percentages of T > 16 mg/L for the calculated unbound fraction of piperacillin.
- In patients with causative strains only sensitive to PIP-TAZ of all antibiotics given to the patient, piperacillin levels negatively correlated with CRP concentrations of day 4 (*p* < 0.05).

## Additional files

Additional file 1: Piperacillin serum concentrations of all patients. A figure showing the piperacillin serum concentrations of all patients over the course of 4 days. Piperacillin concentrations from two to three administrations on day 1 and from one administration on day 2–4 are presented. Single points always represent trough levels; ^1^patient number of each subfigure. (PPTX 838 kb) Additional file 2: Piperacillin trough levels in relation to creatinine clearance for patients who received 4.5 g piperacillin-tazobactam three times daily. A figure showing piperacillin levels in relation to creatinine clearance. Only patients without use of renal replacement therapy are shown. The second trough level, if available, is presented per day and per patient. (PPTX 51 kb) Additional file 3: Distribution of percentage of time with piperacillin values > 90 mg/L. A figure showing the percentage of target attainment of target 2 (≥50 % > 90 mg/L) in different patient subgroups. Percentage of time of piperacillin > 90 mg/L (a) for all patients, (b) for patients receiving piperacillin-tazobactam three times daily and (c) for patients receiving piperacillin-tazobactam two times daily because of impaired renal function are shown. Boxplots represent medians and interquartile ranges, the ends of the whiskers represent the 5th and 95th percentiles. (PPTX 1153 kb) Additional file 4: Renal replacement therapy and dosage of piperacillin-tazobactam A table showing the type of renal replacement therapy and the daily dosage for each patient. CVVHD, continuous veno-venous hemodialysis; CVVH, continuous veno-venous hemofiltration; CVVHDF, continuous veno-venous hemodiafiltration; IHD, intermittent hemodialysis; TID, piperacillin-tazobactam 4.5 g three times daily; BID, piperacillin-tazobactam 4.5 g twice daily. (DOCX 15 kb) Additional file 5: Target attainment in dependence of subgroups. A table showing the target attainment of values in dependence of actual piperacillin-tazobactam dosage, creatinine clearance, and use of renal replacement therapy. From each patient, one value was evaluated per day in the corresponding subgroup (each day grouped in dependence of the actual creatinine clearance, dosage of piperacillin, or actual use of renal replacement therapy). All values of all days of the different subgroups were evaluated simultaneously. TID, piperacillin-tazobactam 4.5 g three times daily; BID, piperacillin-tazobactam 4.5 g twice daily; CrCl creatinine clearance; RRT, renal replacement therapy. ^1^The second trough levels, if available, was evaluated per day and per patient; ^2^values from the first to the second piperacillin-tazobactam administration were used, if available, per day and per patient; ^3^this subgroup included values of the two patients with the highest body mass index, i.e., 37 and 41 kg/m^2^. (DOCX 15 kb)

## Abbreviations

%T > 90 mg/L: percentage of time >90 mg/L
%T > MIC: time above the minimal inhibitory concentration
BID: twice daily
CRP: C-reactive protein
CV: coefficient of variations
fT > MIC: time above the minimal inhibitory concentration for the unbound antibiotic
ICU: intensive care unit
IQR: interquartile range
PIP: piperacillin
RRT: renal replacement therapy
TAZ: tazobactam
TDM: therapeutic drug monitoring
TID: three times daily
UHPLC-MS/MS: ultra-high-performance liquid chromatography/tandem mass spectrometry

## References

[1] Dellinger RP, Levy MM, Rhodes A, Annane D, Gerlach H, Opal SM (2013). Surviving Sepsis Campaign: international guidelines for management of severe sepsis and septic shock, 2012. Intensive Care Med.

[2] Abdul-Aziz MH, Lipman J, Mouton JW, Hope WW, Roberts JA (2015). Applying pharmacokinetic/pharmacodynamic principles in critically ill patients: optimizing efficacy and reducing resistance development. Semin Respir Crit Care Med.

[3] Felton TW, Goodwin J, O’Connor L, Sharp A, Gregson L, Livermore J (2013). Impact of Bolus dosing versus continuous infusion of Piperacillin and Tazobactam on the development of antimicrobial resistance in Pseudomonas aeruginosa. Antimicrob Agents Chemother.

[4] Magill SS, Edwards JR, Beldavs ZG, Dumyati G, Janelle SJ, Kainer MA (2014). Prevalence of antimicrobial use in US acute care hospitals, May-September 2011. JAMA.

[5] Meyer E. Surveillance der Antibiotika-Anwendung und der bakteriellen Resistenzen auf Intensivstationen. 2015. http://sari.eu-burden.info/auswertung/down/AD-ZEIT.pdf. Accessed 26 Nov 2015. 10.1007/s00103-008-0614-6 18787871

[6] Carlier M, Carrette S, Stove V, Verstraete AG, De Waele JJ (2014). Does consistent piperacillin dosing result in consistent therapeutic concentrations in critically ill patients? A longitudinal study over an entire antibiotic course. Int J Antimicrob Agents.

[7] Felton TW, Hope WW, Lomaestro BM, Butterfield JM, Kwa AL, Drusano GL (2012). Population pharmacokinetics of extended-infusion piperacillin-tazobactam in hospitalized patients with nosocomial infections. Antimicrob Agents Chemother.

[8] Gonçalves-Pereira J, Póvoa P (2011). Antibiotics in critically ill patients: a systematic review of the pharmacokinetics of β-lactams. Crit Care.

[9] Roberts DM, Roberts JA, Roberts MS, Liu X, Nair P, Cole L (2012). Variability of antibiotic concentrations in critically ill patients receiving continuous renal replacement therapy: a multicentre pharmacokinetic study. Crit Care Med.

[10] Sime FB, Roberts MS, Peake SL, Lipman J, Roberts JA (2012). Does beta-lactam pharmacokinetic variability in critically ill patients justify therapeutic drug monitoring? A systematic review. Ann Intensive Care.

[11] Felton TW, Roberts JA, Lodise TP, Van Guilder M, Boselli E, Neely MN (2014). Individualization of piperacillin dosing for critically ill patients: dosing software to optimize antimicrobial therapy. Antimicrob Agents Chemother.

[12] Lodise TP, Lomaestro B, Rodvold KA, Danziger LH, Drusano GL (2004). Pharmacodynamic profiling of piperacillin in the presence of tazobactam in patients through the use of population pharmacokinetic models and Monte Carlo simulation. Antimicrob Agents Chemother.

[13] Roberts JA, Paul SK, Akova M, Bassetti M, De Waele JJ, Dimopoulos G (2014). DALI: defining antibiotic levels in intensive care unit patients: are current β-lactam antibiotic doses sufficient for critically ill patients?. Clin Infect Dis.

[14] Udy AA, Lipman J, Jarrett P, Klein K, Wallis SC, Patel K (2015). Are standard doses of piperacillin sufficient for critically ill patients with augmented creatinine clearance?. Crit Care.

[15] Conil JM, Georges B, Mimoz O, Dieye E, Ruiz S, Cougot P (2006). Influence of renal function on trough serum concentrations of piperacillin in intensive care unit patients. Intensive Care Med.

[16] Dalhoff K, Abele-Horn M, Andreas S, Bauer T, von Baum H, Deja M (2012). Epidemiology, diagnosis and treatment of adult patients with nosocomial pneumonia. S-3 Guideline of the German Society for Anaesthesiology and Intensive Care Medicine, the German Society for Infectious Diseases, the German Society for Hygiene and Microbiology, the German Respiratory Society and the Paul-Ehrlich-Society for Chemotherapy. Pneumologie.

[17] Zander J, Maier B, Suhr A, Zoller M, Frey L, Teupser D (2015). Quantification of piperacillin, tazobactam, cefepime, meropenem, ciprofloxacin and linezolid in serum using an isotope dilution UHPLC-MS/MS method with semi-automated sample preparation. Clin Chem Lab Med.

[18] The European Committee on Antimicrobial Susceptibility Testing. Breakpoint tables for interpretation of MICs and zone diameters. Version 5.0, 2015. http://www.eucast.org. Accessed 27 Sept 2015.

[19] Jamal JA, Roberts DM, Udy AA, Mat-Nor MB, Mohamad-Nor FS, Wallis SC (2015). Pharmacokinetics of piperacillin in critically ill patients receiving continuous venovenous haemofiltration: a randomised controlled trial of continuous infusion versus intermittent bolus administration. Int J Antimicrob Agents.

[20] Piperacillin and Tazobactam. http://www.drugs.com/pro/piperacillin-and-tazobactam.html. Accessed 27 Aug 2015.

[21] Sime FB, Roberts MS, Warner MS, Hahn U, Robertson TA, Yeend S (2014). Altered pharmacokinetics of piperacillin in febrile neutropenic patients with hematological malignancy. Antimicrob Agents Chemother.

[22] Wong G, Briscoe S, Adnan S, McWhinney B, Ungerer J, Lipman J (2013). Protein binding of β-lactam antibiotics in critically ill patients: can we successfully predict unbound concentrations?. Antimicrob Agents Chemother.

[23] Taccone FS, Laterre PF, Dugernier T, Spapen H, Delattre I, Wittebole X (2010). Insufficient β-lactam concentrations in the early phase of severe sepsis and septic shock. Crit Care.

[24] Asín-Prieto E, Rodríguez-Gascón A, Trocóniz IF, Soraluce A, Maynar J, Sánchez-Izquierdo JÁ (2014). Population pharmacokinetics of piperacillin and tazobactam in critically ill patients undergoing continuous renal replacement therapy: application to pharmacokinetic/pharmacodynamic analysis. J Antimicrob Chemother.

[25] Carlier M, Carrette S, Roberts JA, Stove V, Verstraete A, Hoste E (2013). Meropenem and piperacillin/tazobactam prescribing in critically ill patients: does augmented renal clearance affect pharmacokinetic/pharmacodynamic target attainment when extended infusions are used?. Crit Care.

[26] Casu GS, Hites M, Jacobs F, Cotton F, Wolff F, Beumier M (2013). Can changes in renal function predict variations in β-lactam concentrations in septic patients?. Int J Antimicrob Agents.

[27] Patel N, Scheetz MH, Drusano GL, Lodise TP (2010). Determination of antibiotic dosage adjustments in patients with renal impairment: elements for success. J Antimicrob Chemother.

[28] Patel N, Scheetz MH, Drusano GL, Lodise TP (2010). Identification of optimal renal dosage adjustments for traditional and extended-infusion piperacillin-tazobactam dosing regimens in hospitalized patients. Antimicrob Agents Chemother.

[29] Udy AA, Varghese JM, Altukroni M, Briscoe S, McWhinney BC, Ungerer JP (2012). Subtherapeutic initial β-lactam concentrations in select critically ill patients: association between augmented renal clearance and low trough drug concentrations. Chest.

[30] Beumier M, Casu GS, Hites M, Seyler L, Cotton F, Vincent JL (2014). β-lactam antibiotic concentrations during continuous renal replacement therapy. Crit Care.

[31] Blondiaux N, Wallet F, Favory R, Onimus T, Nseir S, Courcol RJ (2010). Daily serum piperacillin monitoring is advisable in critically ill patients. Int J Antimicrob Agents.

[32] Huttner A, Von Dach E, Renzoni A, Huttner BD, Affaticati M, Pagani L (2015). Augmented renal clearance, low β-lactam concentrations and clinical outcomes in the critically ill: an observational prospective cohort study. Int J Antimicrob Agents.

[33] Varghese JM, Jarrett P, Boots RJ, Kirkpatrick CM, Lipman J, Roberts JA (2014). Pharmacokinetics of piperacillin and tazobactam in plasma and subcutaneous interstitial fluid in critically ill patients receiving continuous venovenous haemodiafiltration. Int J Antimicrob Agents.

[34] Roberts JA, Abdul-Aziz MH, Lipman J, Mouton JW, Vinks AA, Felton TW (2014). Individualised antibiotic dosing for patients who are critically ill: challenges and potential solutions. Lancet Infect Dis.

[35] Turnidge JD (1998). The pharmacodynamics of beta-lactams. Cin Infect Dis.

[36] Li C, Du X, Kuti JL, Nicolau DP (2007). Clinical pharmacodynamics of meropenem in patients with lower respiratory tract infections. Antimicrob Agents Chemother.

[37] Goncalves-Pereira J, Paiva JA (2013). Dose modulation: a new concept of antibiotic therapy in the critically ill patient?. J Crit Care.

[38] Chatellier D, Jourdain M, Mangalaboyi J, Ader F, Chopin C, Derambure P (2002). Cefepime-induced neurotoxicity: an underestimated complication of antibiotherapy in patients with acute renal failure. Intensive Care Med.

[39] Abdul-Aziz MH, Lipman J, Akova M, Bassetti M, De Waele JJ, Dimopoulos G (2016). Is prolonged infusion of piperacillin/tazobactam and meropenem in critically ill patients associated with improved pharmacokinetic/pharmacodynamic and patient outcomes? An observation from the Defining Antibiotic Levels in Intensive care unit patients (DALI) cohort. J Antimicrob Chemother.

[40] Dulhunty JM, Roberts JA, Davis JS, Webb SA, Bellomo R, Gomersall C (2015). A multicenter randomized trial of continuous versus intermittent β-lactam infusion in severe sepsis. Am J Respir Crit Care Med.

[41] Roberts JA, Webb S, Paterson D, Ho KM, Lipman J (2009). A systematic review on clinical benefits of continuous administration of beta-lactam antibiotics. Crit Care Med.

[42] Charles B (2014). Population pharmacokinetics: an overview. Aust Prescr.

