# Supplementary material for: Piperacillin concentration in relation to therapeutic range in critically ill patients – a prospective observational study
Source: Crit Care. 2016 Apr 4;20:79. doi: 10.1186/s13054-016-1255-z (PMC4819271; doi:10.1186/s13054-016-1255-z)
Supplement: Additional file 1: — Piperacillin serum concentrations of all patients. A figure showing the piperacillin serum concentrations of all patients over the course of 4 days. Piperacillin concentrations from two to three administrations on day 1 and from one administration on day 2–4 are presented. Single points always represent trough levels; 1patient number of each subfigure. (PPTX 838 kb) [file 13054_2016_1255_MOESM1_ESM.pptx]

## Slide 1
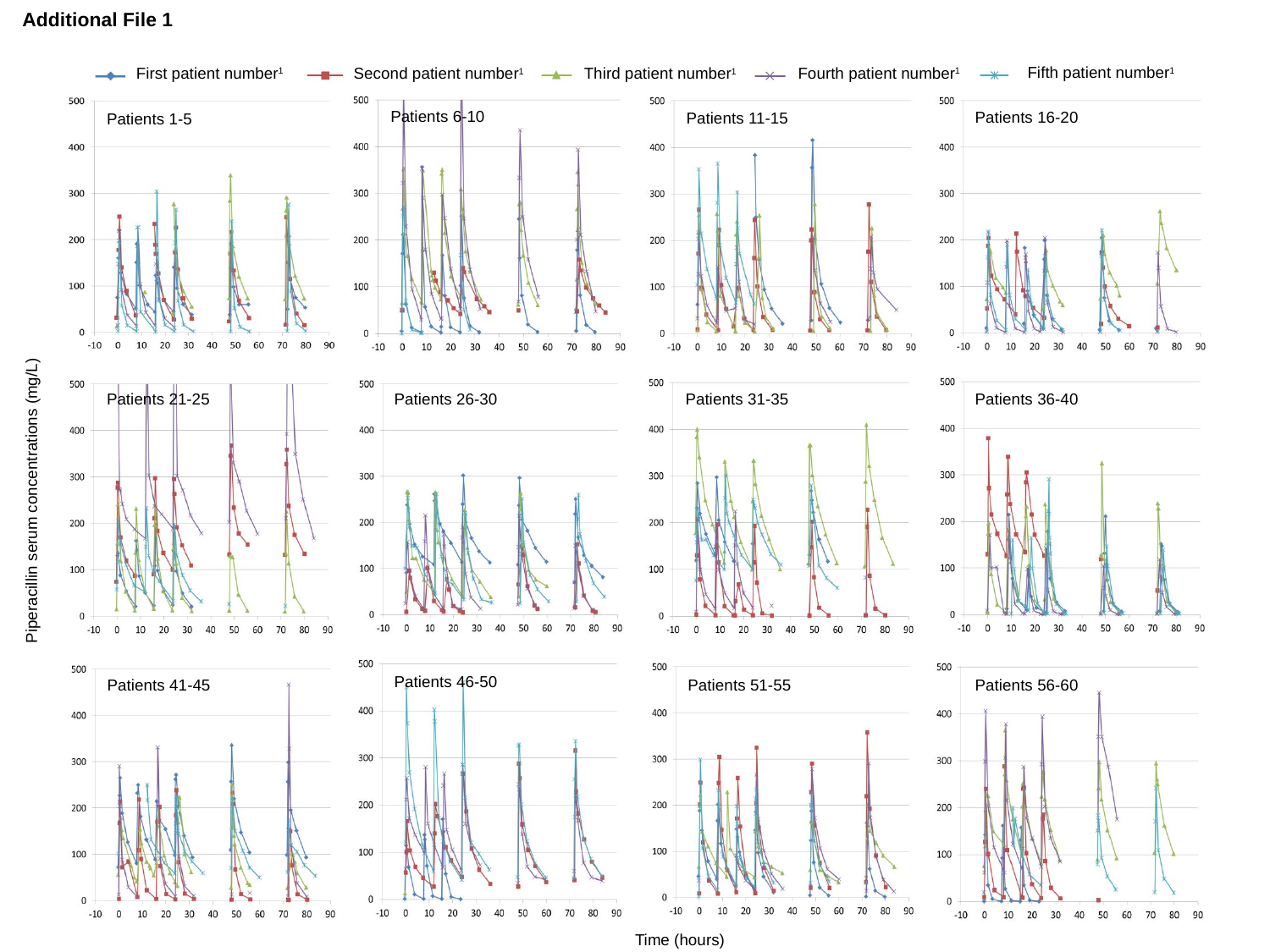

Additional File 1
Fifth patient number1
First patient number1
Fourth patient number1
Second patient number1
Third patient number1
Patients 6-10
Patients 16-20
Patients 11-15
Patients 1-5
Patients 21-25
Patients 36-40
Patients 26-30
Patients 31-35
Piperacillin serum concentrations (mg/L)
Patients 46-50
Patients 41-45
Patients 51-55
Patients 56-60
Time (hours)
