# Supplementary material for: Piperacillin concentration in relation to therapeutic range in critically ill patients – a prospective observational study
Source: Crit Care. 2016 Apr 4;20:79. doi: 10.1186/s13054-016-1255-z (PMC4819271; doi:10.1186/s13054-016-1255-z)
Supplement: Additional file 2: — Piperacillin trough levels in relation to creatinine clearance for patients who received 4.5 g piperacillin-tazobactam three times daily. A figure showing piperacillin levels in relation to creatinine clearance. Only patients without use of renal replacement therapy are shown. The second trough level, if available, is presented per day and per patient. (PPTX 51 kb) [file 13054_2016_1255_MOESM2_ESM.pptx]

## Slide 1
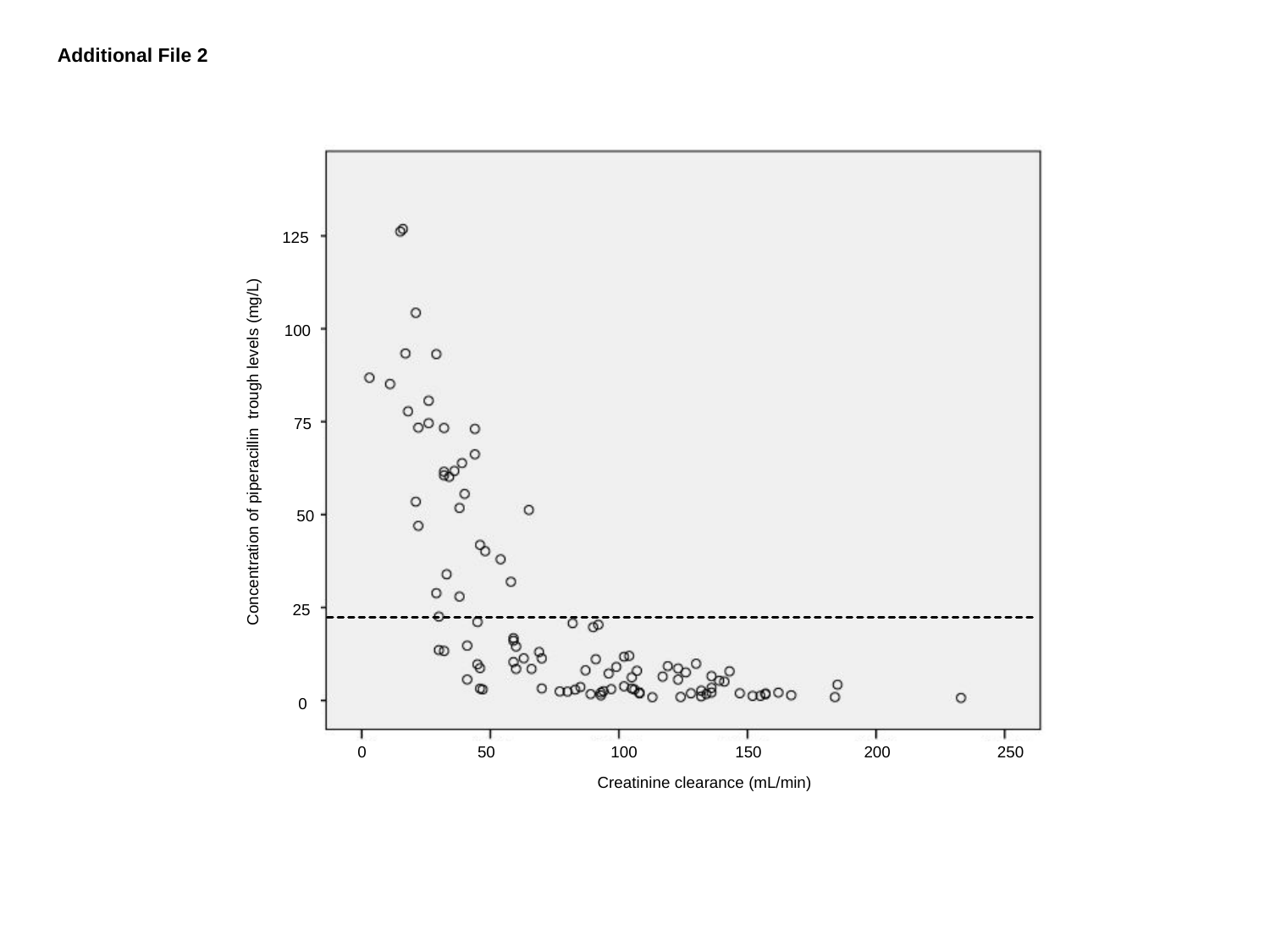

Additional File 2
125
100
Concentration of piperacillin trough levels (mg/L)
 75
50
 25
 0
 0 50 100 150 200 250
Creatinine clearance (mL/min)
