# Supplementary material for: Piperacillin concentration in relation to therapeutic range in critically ill patients – a prospective observational study
Source: Crit Care. 2016 Apr 4;20:79. doi: 10.1186/s13054-016-1255-z (PMC4819271; doi:10.1186/s13054-016-1255-z)
Supplement: Additional file 3: — Distribution of percentage of time with piperacillin values > 90 mg/L. A figure showing the percentage of target attainment of target 2 (≥50 % > 90 mg/L) in different patient subgroups. Percentage of time of piperacillin > 90 mg/L (a) for all patients, (b) for patients receiving piperacillin-tazobactam three times daily and (c) for patients receiving piperacillin-tazobactam two times daily because of impaired renal function are shown. Boxplots represent medians and interquartile ranges, the ends of the whiskers represent the 5th and 95th percentiles. (PPTX 1153 kb) [file 13054_2016_1255_MOESM3_ESM.pptx]

## Slide 1
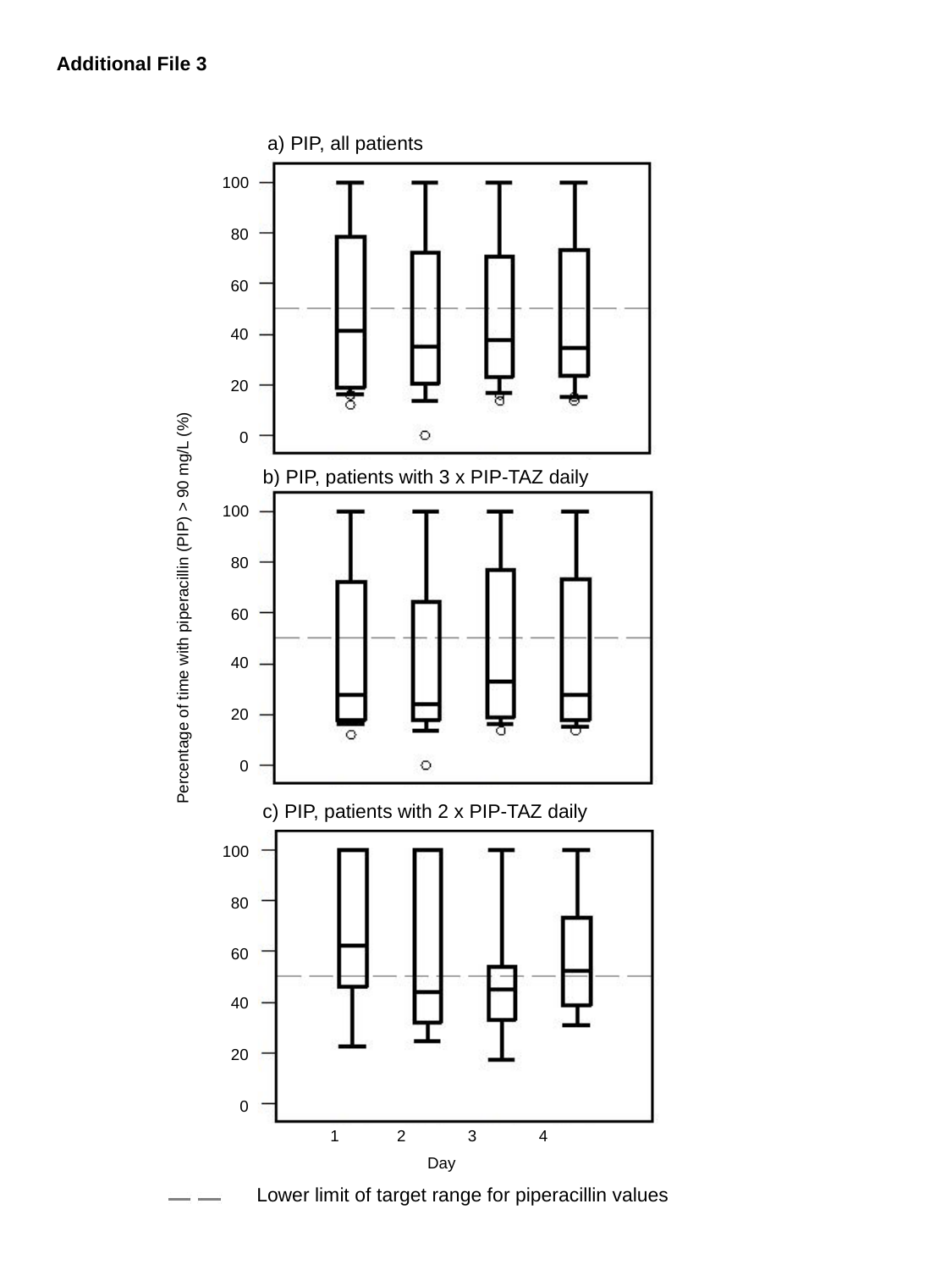

Additional File 3
a) PIP, all patients
100
 80
 60
 40
 20
 0
b) PIP, patients with 3 x PIP-TAZ daily
100
 80
Percentage of time with piperacillin (PIP) > 90 mg/L (%)
 60
 40
 20
 0
c) PIP, patients with 2 x PIP-TAZ daily
100
 80
 60
 40
 20
 0
1 2 3 4
Day
Lower limit of target range for piperacillin values
