# Supplementary material for: Piperacillin concentration in relation to therapeutic range in critically ill patients – a prospective observational study
Source: Crit Care. 2016 Apr 4;20:79. doi: 10.1186/s13054-016-1255-z (PMC4819271; doi:10.1186/s13054-016-1255-z)
Supplement: Additional file 4: — Renal replacement therapy and dosage of piperacillin-tazobactam A table showing the type of renal replacement therapy and the daily dosage for each patient. CVVHD, continuous veno-venous hemodialysis; CVVH, continuous veno-venous hemofiltration; CVVHDF, continuous veno-venous hemodiafiltration; IHD, intermittent hemodialysis; TID, piperacillin-tazobactam 4.5 g three times daily; BID, piperacillin-tazobactam 4.5 g twice daily. (DOCX 15 kb) [file 13054_2016_1255_MOESM4_ESM.docx]

**Additional File 4: Renal replacement therapy and dosage of piperacillin-tazobactam**

| **Patient number** | **Day 1** | **Day 2** | **Day 3** | **Day 4** |
| --- | --- | --- | --- | --- |
| 2 | CVVHD  TID | CVVHD  TID | -  TID | CVVHD  TID |
| 14 | CVVHD  TID | CVVHD  TID | CVVHD  TID | CVVHD  BID |
| 18 | CVVH  TID | CVVHD  TID | CVVHD  TID | -  TID |
| 22 | -  TID | CVVHD  TID | CVVHD  TID | CVVHD  TID |
| 23 | CVVHD  TID | CVVHD  TID | CVVHD  TID | CVVHD  TID |
| 26 | -  BID | -  BID | -  BID | CVVHD  BID |
| 28 | CVVHD  BID | CVVHD  BID | CVVHD  BID |  |
| 30 | -  BID | -  BID | CVVHDF  BID | -  BID |
| 31 | CVVHDF  TID | -  TID | CVVHD  TID |  |
| 35 | -  BID | -  BID | IHD  BID | -  BID |
| 45 | IHD  BID | -  BID | IHD  BID | -  BID |
| 49 | -  TID | CVVHD  TID | CVVHD  BID | -  BID |
| 50 | CVVHD  BID | CVVHD  BID | CVVHD  BID | CVVHD  BID |
| 53 | -  BID | CVVH  BID | -  BID | -  BID |
| 58 | CVVHDF  TID | CVVHDF  TID | CVVHDF  TID | CVVHDF  TID |
| 60 | CVVHD  TID | CVVHD  TID | CVVHD  TID | -  TID |

CVVHD, Continuous veno-venous hemodialysis; CVVH, Continuous veno-venous hemofiltration; CVVHDF, Continuous veno-venous hemodiafiltration; IHD, intermittent haemodialysis; TID, Three times daily piperacillin-tazobactam 4.5 g; BID, Two times daily piperacillin-tazobactam 4.5 g
