# Supplementary material for: Piperacillin concentration in relation to therapeutic range in critically ill patients – a prospective observational study
Source: Crit Care. 2016 Apr 4;20:79. doi: 10.1186/s13054-016-1255-z (PMC4819271; doi:10.1186/s13054-016-1255-z)
Supplement: Additional file 5: — Target attainment in dependence of subgroups. A table showing the target attainment of values in dependence of actual piperacillin-tazobactam dosage, creatinine clearance, and use of renal replacement therapy. From each patient, one value was evaluated per day in the corresponding subgroup (each day grouped in dependence of the actual creatinine clearance, dosage of piperacillin, or actual use of renal replacement therapy). All values of all days of the different subgroups were evaluated simultaneously. TID, piperacillin-tazobactam 4.5 g three times daily; BID, piperacillin-tazobactam 4.5 g twice daily; CrCl creatinine clearance; RRT, renal replacement therapy. 1The second trough levels, if available, was evaluated per day and per patient; 2values from the first to the second piperacillin-tazobactam administration were used, if available, per day and per patient; 3this subgroup included values of the two patients with the highest body mass index, i.e., 37 and 41 kg/m2. (DOCX 15 kb) [file 13054_2016_1255_MOESM5_ESM.docx]

**Additional File 5: Target attainment in dependence of subgroups**

| **Subgroup characteristics** | **Percentages of values that attain the targets over the four study days** | |
| --- | --- | --- |
|  | **trough values^1^  ≥ 22.5 mg/L** | **≥ 50% of time  > 90 mg/L^2^** |
| TID, RRT | 76^1^ | 52^1^ |
| TID, CrCl < 30 mL/min | 100 | 92 |
| TID, CrCl 30-65 mL/min | 57 | 37 |
| TID, CrCl > 65 mL/min | 0 | 6 |
|  |  |  |
| BID, RRT | 100 | 57 |
| BID, CrCl < 30 mL/min | 91 | 45 |
| BID, CrCl 30-65 mL/min | 50 | 0 |

From each patient, one value was evaluated per day in the corresponding subgroup (each day grouped in dependence of the actual creatinine clearance, dosage of piperacillin, or actual use of RRT). All values of all days of the different subgroups were evaluated simultaneously.

TID, Three times daily piperacillin-tazobactam 4.5 g; BID, Two times daily piperacillin-tazobactam 4.5 g; CrCl creatinine clearance; RRT, renal replacement therapy

^1^, The second trough levels, if available, was evaluated per day and per patient; ^2^, Values from the first to the second piperacillin-tazobactam administration were used, if available, per day and per patient; ^3^, This subgroup included values of the two patients with the highest body mass index, i.e., 37 and 41 kg/m²
